# High expression of TARS is associated with poor prognosis of endometrial cancer

**DOI:** 10.18632/aging.204558

**Published:** 2023-03-06

**Authors:** Lihui Si, Lianchang Liu, Ruiqi Yang, Wenxin Li, Xiaohong Xu

**Affiliations:** 1 Department of Obstetrics and Gynecology, The Second Hospital of Jilin University, Changchun 130021, China; 2 Department of Intervention, The Second Hospital of Jilin University, Changchun 130021, China; 3 Physical Examination Center, The Second Hospital of Jilin University, Changchun 130021, China

**Keywords:** TARS, overall survival, disease specific survival, endometrial cancer, biomarker

## Abstract

Introduction: Endometrial cancer is the second largest and most common cancer in the world. It is urgent to explore novel biomarkers.

Methods: Data were obtained from The Cancer Genome Atlas (TCGA) database. The receiver operating characteristic (ROC) curves, Kaplan-Meier curves and Cox analysis, nomograms, gene set enrichment analysis (GSEA) were conducted. Cell proliferation experiments were performed in Ishikawa cell.

Results: TARS was significantly highly expressed in serous type, G3 grade, and deceased status. Significant association was between high TARS expression with poor overall survival (*P* = 0.0012) and poor disease specific survival (*P* = 0.0034). Significant differences were observed in advanced stage, G3 and G4, and old. The stage, diabetes, histologic grade, and TARS expression showed independent prognostic value for overall survival of endometrial cancer. The stage, histologic grade, and TARS expression showed independent prognostic value for disease specific survival of endometrial cancer. Activated CD4^+^ T cell, effector memory CD4^+^ T cell, memory B cell and type 2 T helper cell may participate in the high TARS expression related immune response in endometrial cancer. The CCK-8 results showed significantly inhibited cell proliferation in si-TARS (*P* < 0.05) and promoted cell proliferation in O-TARS (*P* < 0.05), confirmed by the colony formation and live/dead staining.

Conclusion: High TARS expression was found in endometrial cancer with prognostic and predictive value. This study will provide new biomarker TARS for diagnosis and prognosis of endometrial cancer.

## INTRODUCTION

Endometrial cancer is a common cancer in female reproductive systems, and is the second largest and most common cancer in the world second only to cervical cancer [1]. There can be 319,500 new cases in the world each year, and the mortality rate is higher than 23% [2]. The rate of incidence is rising among younger females [3]. Endometrial cancer begins in the endometrium, located at the innermost layer of the uterus and is the result of abnormal growth of cells with invasion or spread to other parts of the body [4]. Endometrial cancer is divided into type I hormone dependencies and type II non-hormone dependencies [5]. Type I endometrium cancer is an endometrium-like adenocarcinoma in tissue classification, which is the most common subtype with good prognosis [6]. Meanwhile, type II endometrial cancer carries mutant genes such as P53, P16, etc., and has a high risk of metastasis and poor prognosis [7].

The most common clinical manifestation of endometrial cancer is abnormal uterine bleeding, but this symptom can also be caused by many other diseases [8]. In some cases, endometrial cancer may have already developed into advanced stage when signs and symptoms can be noticed. At present, the treatment of endometrial cancer is mainly surgery, followed by comprehensive treatment with auxiliary methods such as radiotherapy, chemotherapy, and hormone therapy [9]. However, the local high recurrence rate, high metastasis rate, and hormone therapy resistance are still the predicament of clinical treatment [10]. Therefore, it is urgent to explore the potential molecular mechanism of endometrial cancer progression, and find novel biomarkers and effective treatment targets.

Amino acids are attached to their corresponding tRNAs by enzymes called aminoacyl-tRNA synthetases (ARSs), which play important roles in protein synthesis [11]. The threonyl-tRNA synthetase (TARS) is one of the ARSs and serves as an important therapeutic target [12]. TARS was discovered as an active enzyme in the mid-1950s, which can produce a carboxyl activated complex that combined with enzymes [13]. However, the role of TRAS in endometrial cancer has not been illuminated yet. In this study, we examined the association between clinicopathologic characteristics and TARS expression in endometrial cancer using data from The Cancer Genome Atlas (TCGA) database. The receiver operating characteristic (ROC) curves were plotted to study the diagnostic value of TARS expression. The Kaplan-Meier curves and Cox analysis were used to study the overall survival and disease specific survival. The nomograms were used to study the predictive value of TARS expression. The gene set enrichment analysis (GSEA) was conducted, and high TARS expression-enriched pathways indicated their influence on the immune response and cell proliferation of endometrial cancer.

## MATERIALS AND METHODS

### Data mining

The TCGA database (https://www.cancergenome.nih.gov) was used to get the whole RNA-Seq expression files as well as any related clinical features [14]. The TARS mRNA expression data was converted into RSEM-normalized values using log2 (x + 1). The non-parametric rank sum test was employed to evaluate the levels of TARS mRNA expression. The Wilcoxon rank sum test was used to compare two groups, and the Kruskal-Wallis test was used to compare multiple groups. The Fisher’s exact test and chi-square test were employed to evaluate the association between clinical traits and TARS expression.

### Diagnostic value of TARS expression

Using the pROC application to show ROC curves, we estimated the area under the ROC curves (AUC) values and established the proper cutoff threshold for the evaluation of TARS diagnostic capabilities [15]. The patients were divided into high or low TARS expression according to the cutoff threshold.

### Predictive value of TARS expression

The patients with endometrial cancer were grouped, followed by comparison of histological type, stage, histologic grade, menopause status, and residual tumor for overall survival and disease specific survival.

### GSEA analysis

First, a search of the TCGA database was conducted, and then an online GSEA analysis was performed to look into the relationship between TARS expression and enriched pathways [16].

### Cell culture and plasmid transfection

The human endometrial carcinoma cell line Ishikawa was obtained from Shanghai Biochemical Cell Institute (Shanghai, China). Ishikawa cells were cultured in RPMI 1640 medium, which contains 10% fetal bovine serum and 1% penicillin-streptomycin solution, at 37°C in the 5% CO_2_ humidified atmosphere [17]. The si-TARS and si-control plasmids were purchased from Miaoling Bio (Wuhan, China) for transfection into the cells.

### Real-time quantitative PCR

The total RNA extraction was performed using the Invitrogen kit (Thermo Fisher Scientific, MA, USA), followed by the reverse transcription. The real-time quantitative PCR (qRT-PCR) was conducted for detecting TARS expression. The 2^−ΔΔCt^ approach was used for quantification. The primers were as follows: TARS forward primer, 5′-TGTGTGCCATTGAATAAGGA-3′; TARS reverse primer, 5′-CACCTTCATTATCAAGATAC-3′; β-actin forward primer, ACCCCAAAGCCAACAGA; β-actin reverse primer, CCAGAGTCCATCACAATACC [18].

### Cell proliferation

Plasmids were introduced to the Ishikawa cells and cultured for 24 hours. 10 μL of CCK-8 reagent was added and reacted for 0.5 h. Cell viability was determined using the 490 nm absorbance measurement. The Calcein AM and PI co-staining, as well as colony formation assay, was carried out as previously reported [19].

### Statistical analysis

The analysis was performed using R3.5.1 [20]. The survival rate was examined using the Kaplan-Meier curve [21]. The independent prognostic potential of TARS was examined using univariate and multivariate Cox models. The correlation between TARS expression and immune cells were analyzed. *P* < 0.05 was statistically significant.

## RESULTS

### High TARS expression in endometrial cancer

**Figure 1:**
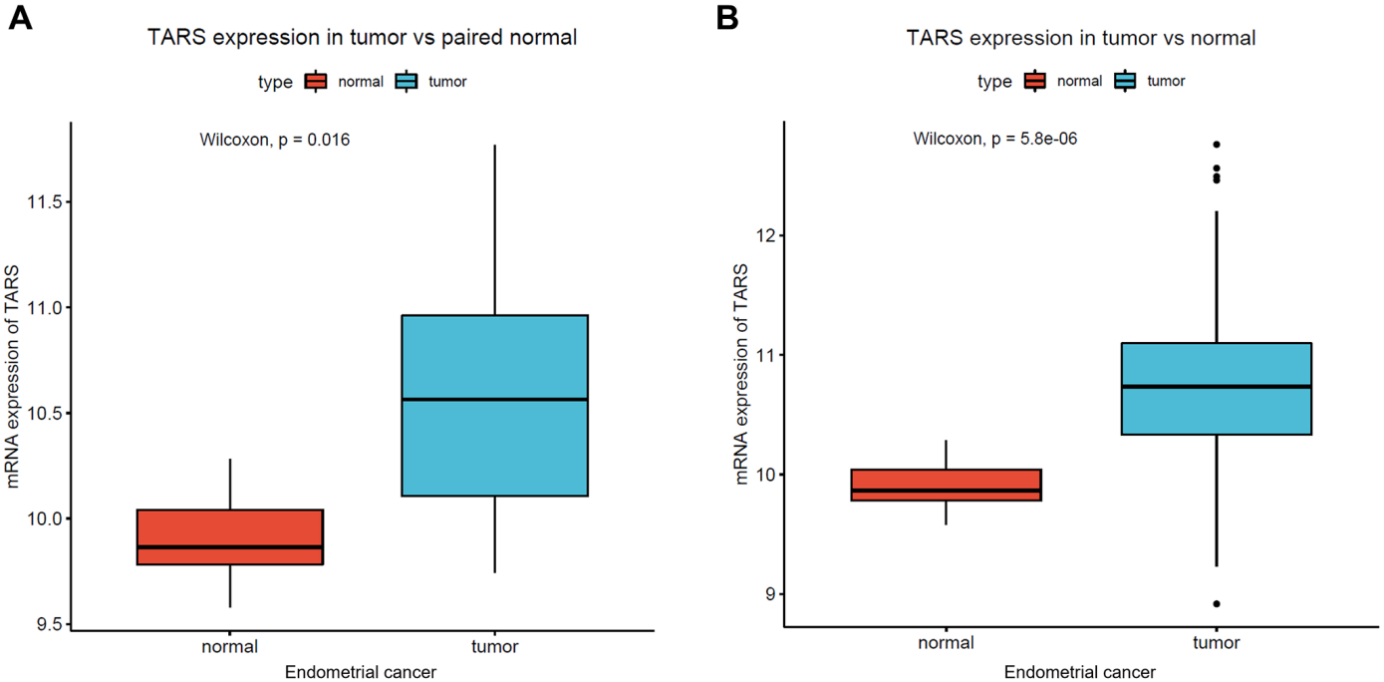
**High TARS expression in endometrial cancer.** (**A**) TARS expression in tumor vs. paired normal tissue. (**B**) TARS expression in tumor vs. normal tissue.

The mRNA expression of TARS was first examined in endometrial cancer. Significant high TARS expression was found in tumor compared with paired normal tissue (*P* = 0.016, Figure 1A), and normal endometrial tissue (*P* < 0.001, Figure 1B).

### Characteristics of patients with endometrial cancer

The characteristics of patients with endometrial cancer were studied using TCGA data. Totally, 370 patients with endometrial cancer were analyzed (Supplementary Table 1). There were 72 patients (19.46%) less than 55 years old. Endometrioid type with 303 patients (81.89%) was most in patients with endometrial cancer. Notably, histologic grade (*P* = 0.0015) and vital status (*P* = 0.0017) were significantly different in high TARS group and low TARS group. Meanwhile, age (*P* = 0.9828), histological type (*P* = 0.5773), stage (*P* = 0.0662), diabetes (*P* = 0.0916), hypertension (*P* = 0.6759), menopause status (*P* = 0.5987), and residual tumor (*P* = 0.5075) showed no statistical differences.

### TARS expression in subgroups

**Figure 2:**
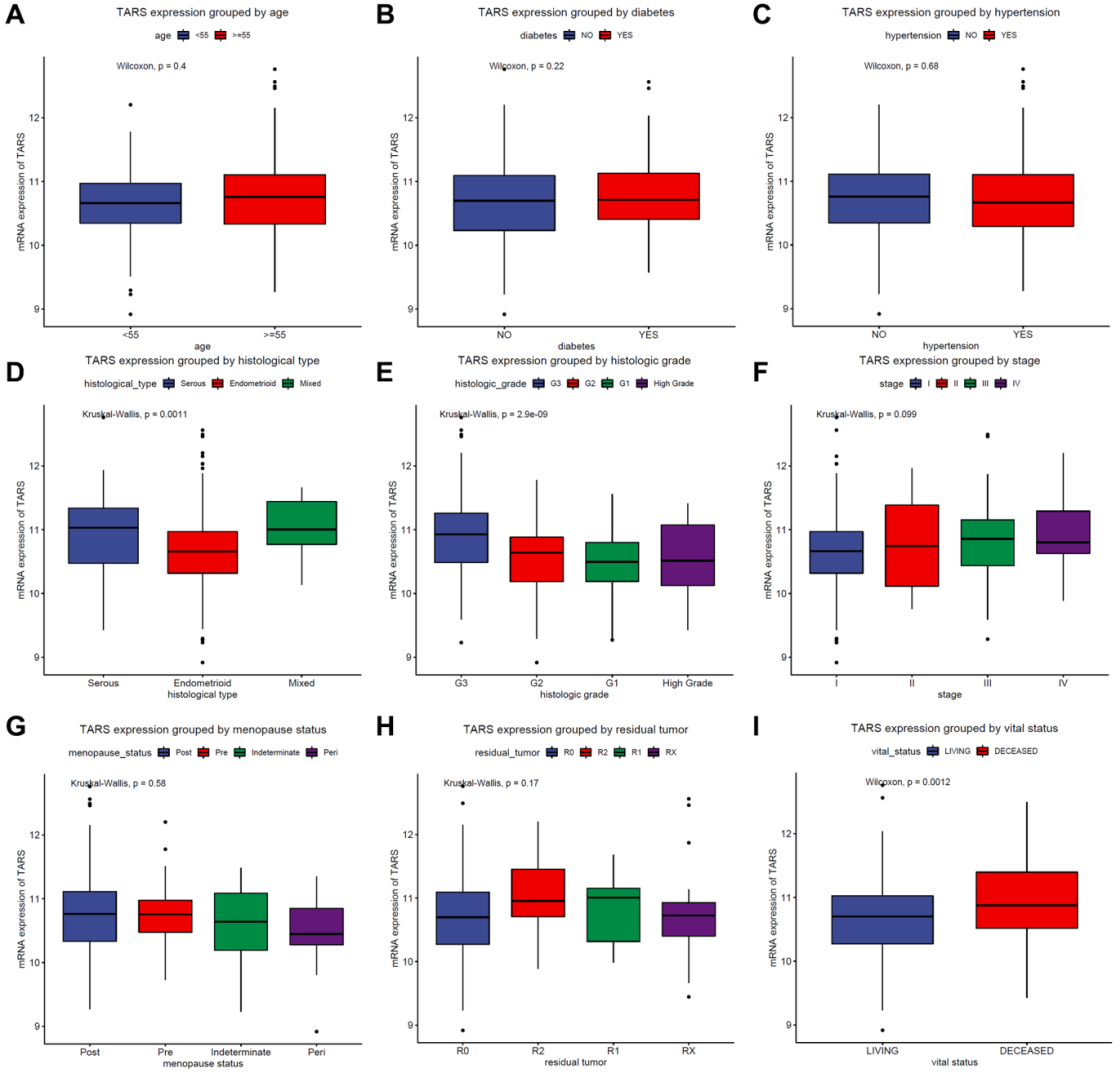
**TARS expression in subgroups.** TARS expression grouped by (**A**) age, (**B**) diabetes, (**C**) hypertension, (**D**) histological type, (**E**) histologic grade, (**F**) stages, (**G**) menopause status, (**H**) residual tumor, and (**I**) vital status.

The TARS expression grouped by age (Figure 2A), diabetes (Figure 2B), hypertension (Figure 2C), histological type (Figure 2D), histologic grade (Figure 2E), stage (Figure 2F), menopause status (Figure 2G), residual tumor (Figure 2H), and vital status (Figure 2I) were exhibited. TARS was significantly highly expressed in serous type (*P* = 0.0011), G3 grade (*P* < 0.001), and deceased status (*P* = 0.0012). However, the other subgroups showed no statistical differences.

### Diagnostic value of TARS expression

The AUC of ROC curve between normal and tumor was 0.901 (Supplementary Figure 1A). Besides, the AUC was 0.890 for stage I (Supplementary Figure 1B), 0.864 for stage II (Supplementary Figure 1C), 0.936 for stage III (Supplementary Figure 1D), and 0.957 for stage IV (Supplementary Figure 1E). The results indicated promising diagnostic value of TARS expression.

### High TARS expression is associated with poor survival

**Figure 3:**
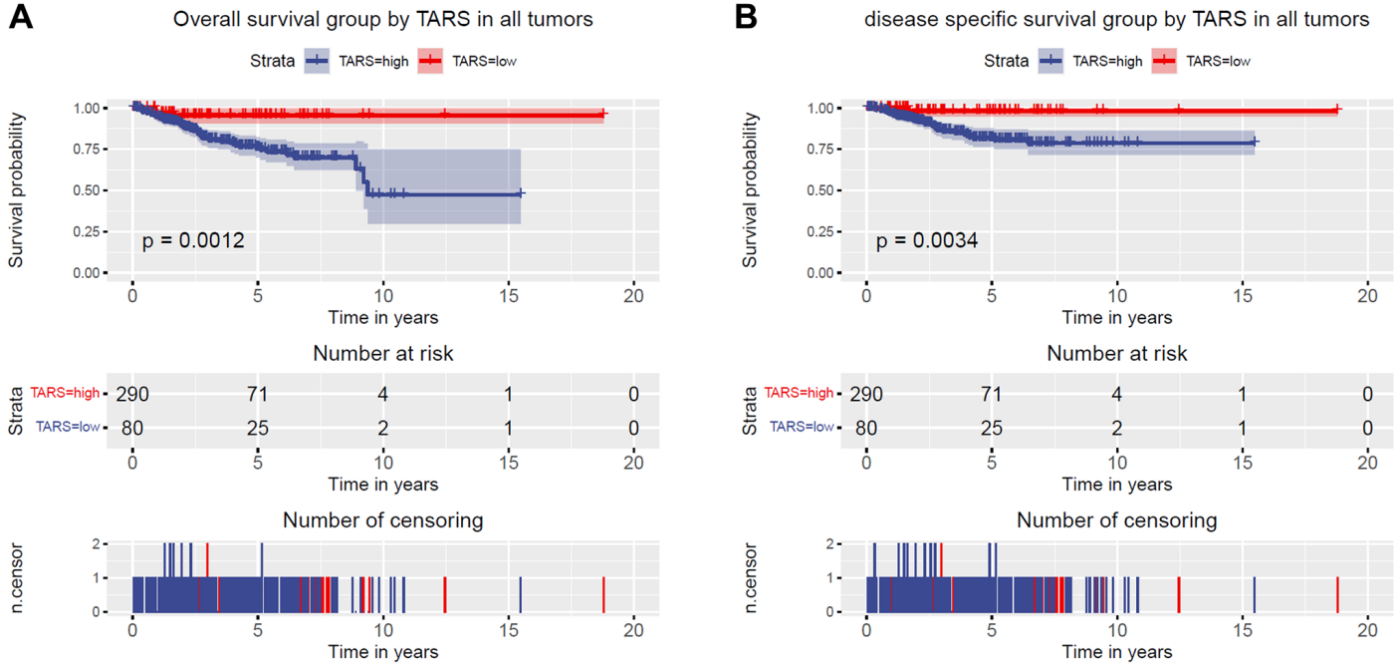
**High TARS expression is associated with poor survival.** (**A**) Overall survival group by TARS in all tumors. (**B**) Disease specific survival group by TARS in all tumors.

Kaplan–Meier curves were plotted to evaluate the overall survival (Figure 3A) and disease specific survival (Figure 3B). The results showed significant association between high TARS expression with poor overall survival (*P* = 0.0012) and poor disease specific survival (*P* = 0.0034).

### Overall survival grouped TARS expression

**Figure 4:**
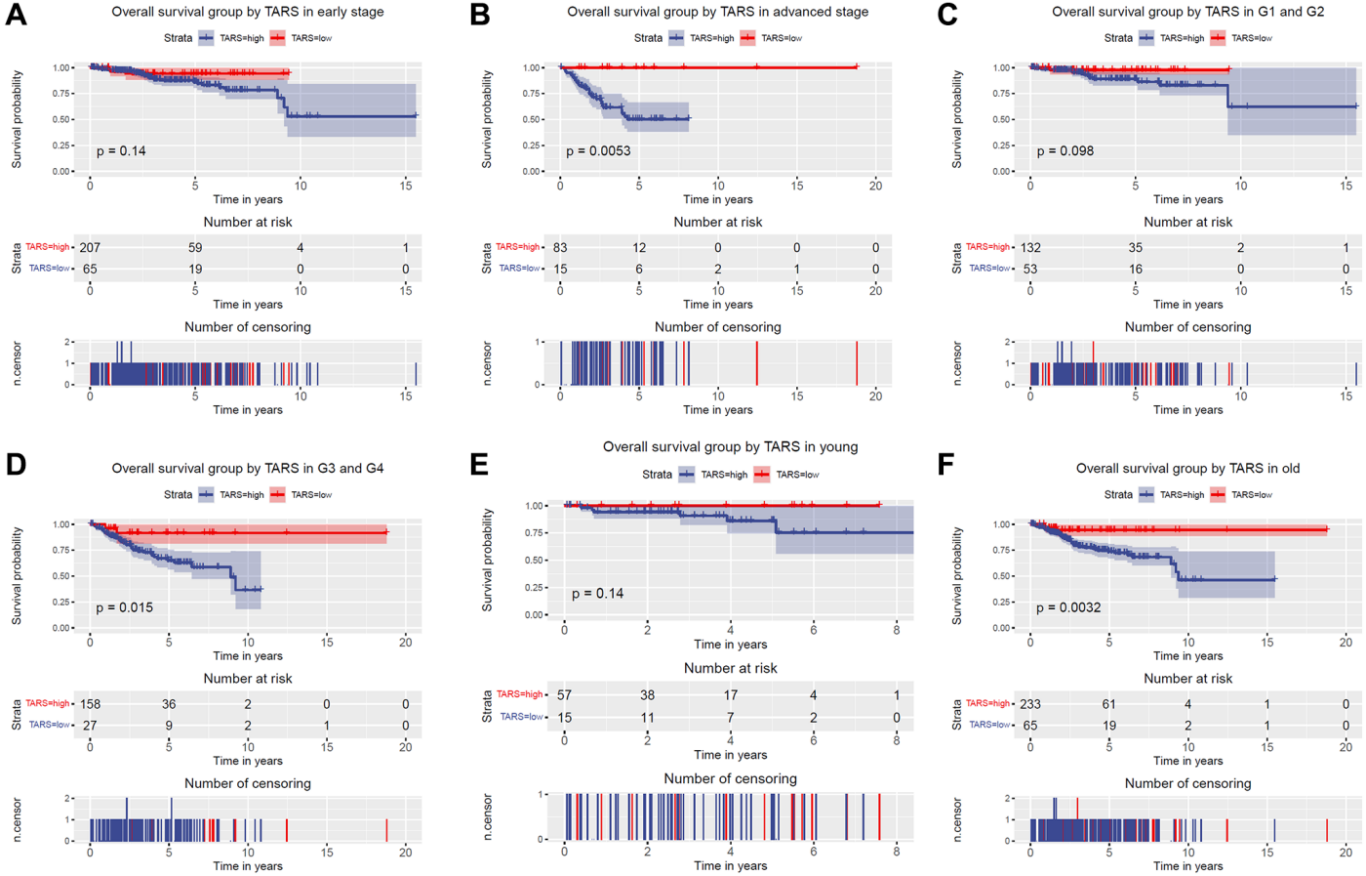
**Overall survival grouped TARS expression.** Overall survival group by GJB3 in (**A**) early stage, (**B**) advanced stage, (**C**) G1 and G2, (**D**) G3 and G4, (**E**) young, and (**F**) old.

The subgroup analysis of overall survival was performed (Figure 4A–4F). Significant differences were observed in advanced stage (*P* = 0.0053), G3 and G4 (*P* = 0.015), and old (*P* = 0.0032). Nevertheless, early stage, G1 and G2, and young subgroups showed no statistical differences.

**Figure 5:**
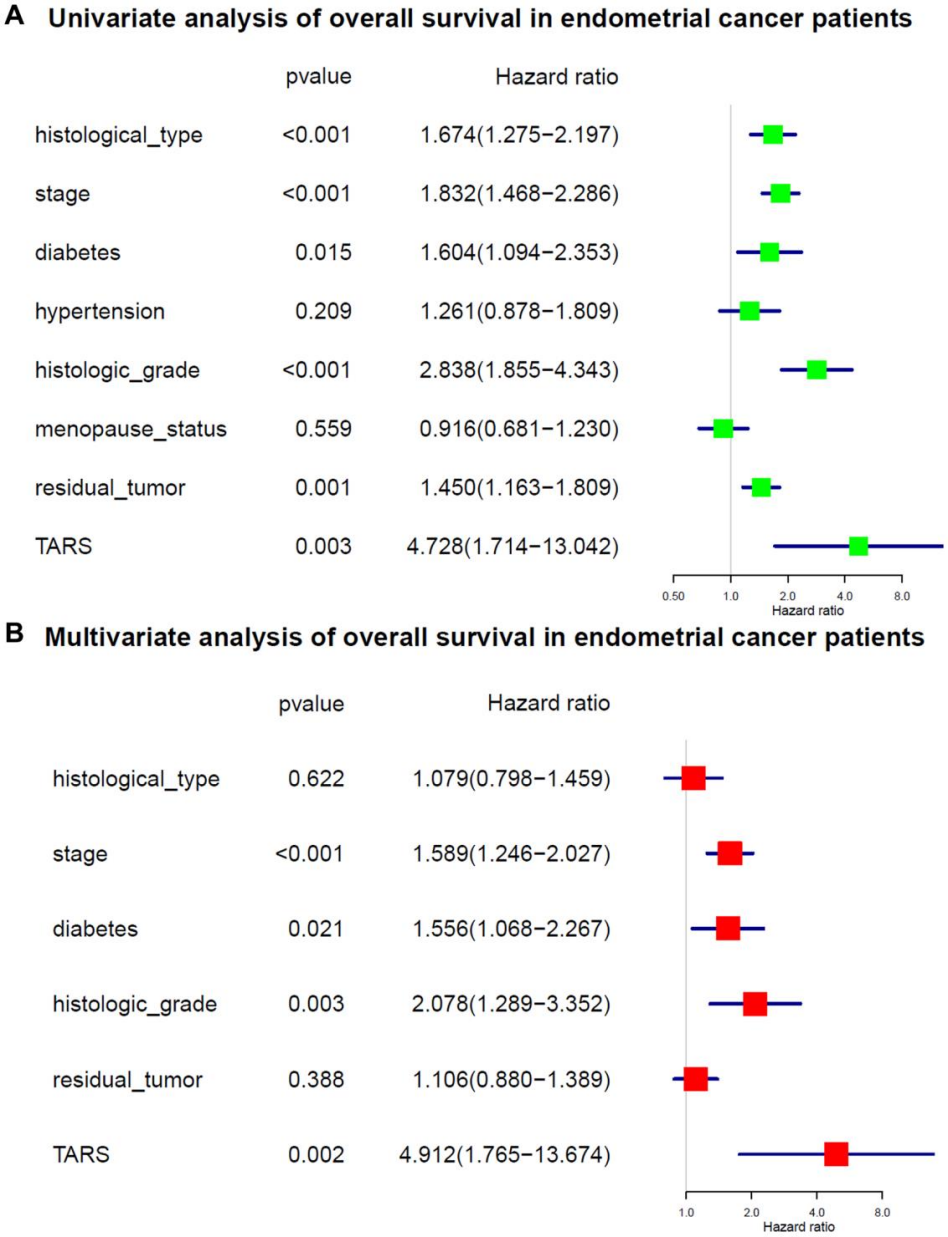
**Cox analysis of overall survival.** (**A**) Univariate analysis of overall survival. (**B**) Multivariate analysis of overall survival.

The variables identified by univariate analysis (Figure 5A) were confirmed by multivariate analysis (Figure 5B). The stage [hazard ratio (HR): 1.589, 95% confidence interval (CI): 1.246–2.027, *P* < 0.001], diabetes (HR: 1.556, 95% CI: 1.068–2.267, *P* = 0.021), histologic grade (HR: 2.078, 95% CI: 1.289–3.352, *P* = 0.003), and TARS expression (HR: 4.912, 95% CI: 1.765–13.674, *P* = 0.002) showed independent prognostic value for overall survival of endometrial cancer.

### Disease specific survival grouped TARS expression

**Figure 6:**
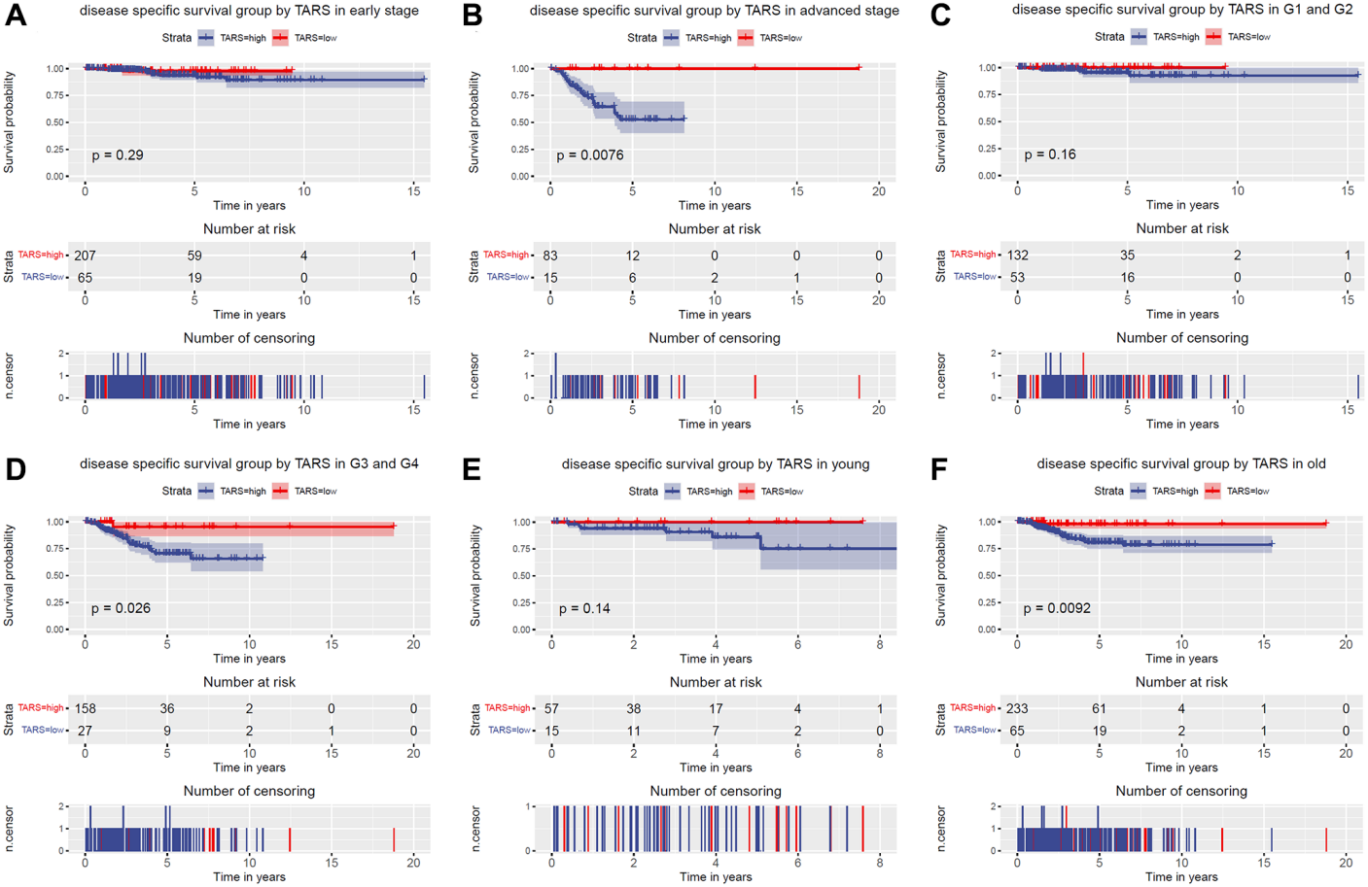
**Disease specific survival grouped TARS expression.** Disease specific survival group by GJB3 in (**A**) early stage, (**B**) advanced stage, (**C**) G1 and G2, (**D**) G3 and G4, (**E**) young, and (**F**) old.

The subgroup analysis of disease specific survival was performed (Figure 6A–6F). Significant differences were observed in advanced stage (*P* = 0.0076), G3 and G4 (*P* = 0.026), and old (*P* = 0.0092). Nevertheless, early stage, G1 and G2, and young subgroups showed no statistical differences.

**Figure 7:**
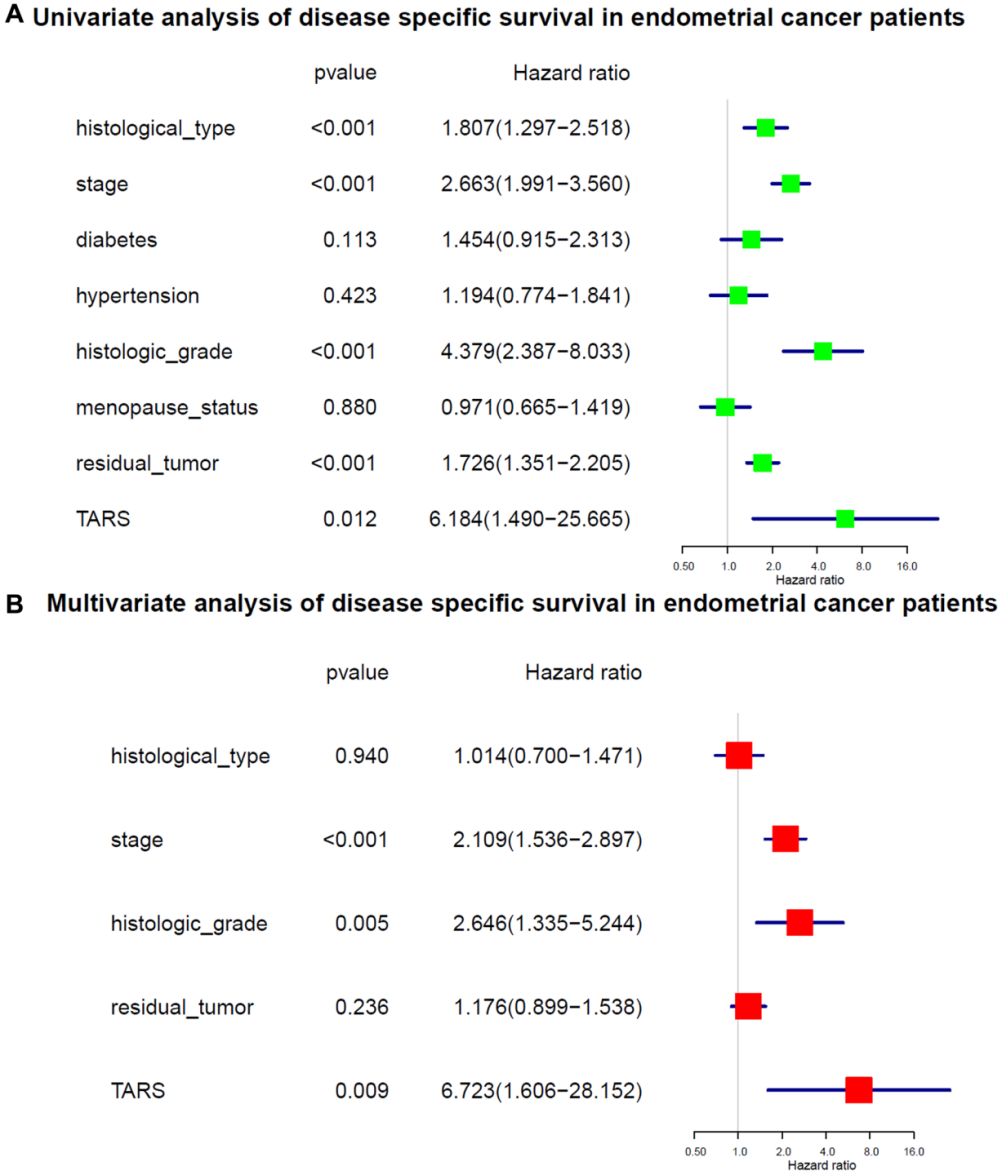
**Cox analysis of disease specific survival.** (**A**) Univariate analysis of disease specific survival. (**B**) Multivariate analysis of disease specific survival.

The variables identified by univariate analysis (Figure 7A) were confirmed by multivariate analysis (Figure 7B). The stage (HR: 2.109, 95% CI: 1.536–2.897, *P* < 0.001), histologic grade (HR: 2.646, 95% CI: 1.335–5.244, *P* = 0.005), and TARS expression (HR: 6.723, 95% CI: 1.606–28.152, *P* = 0.009) showed independent prognostic value for disease specific survival of endometrial cancer.

### Predictive value of TARS expression in overall survival

**Figure 8:**
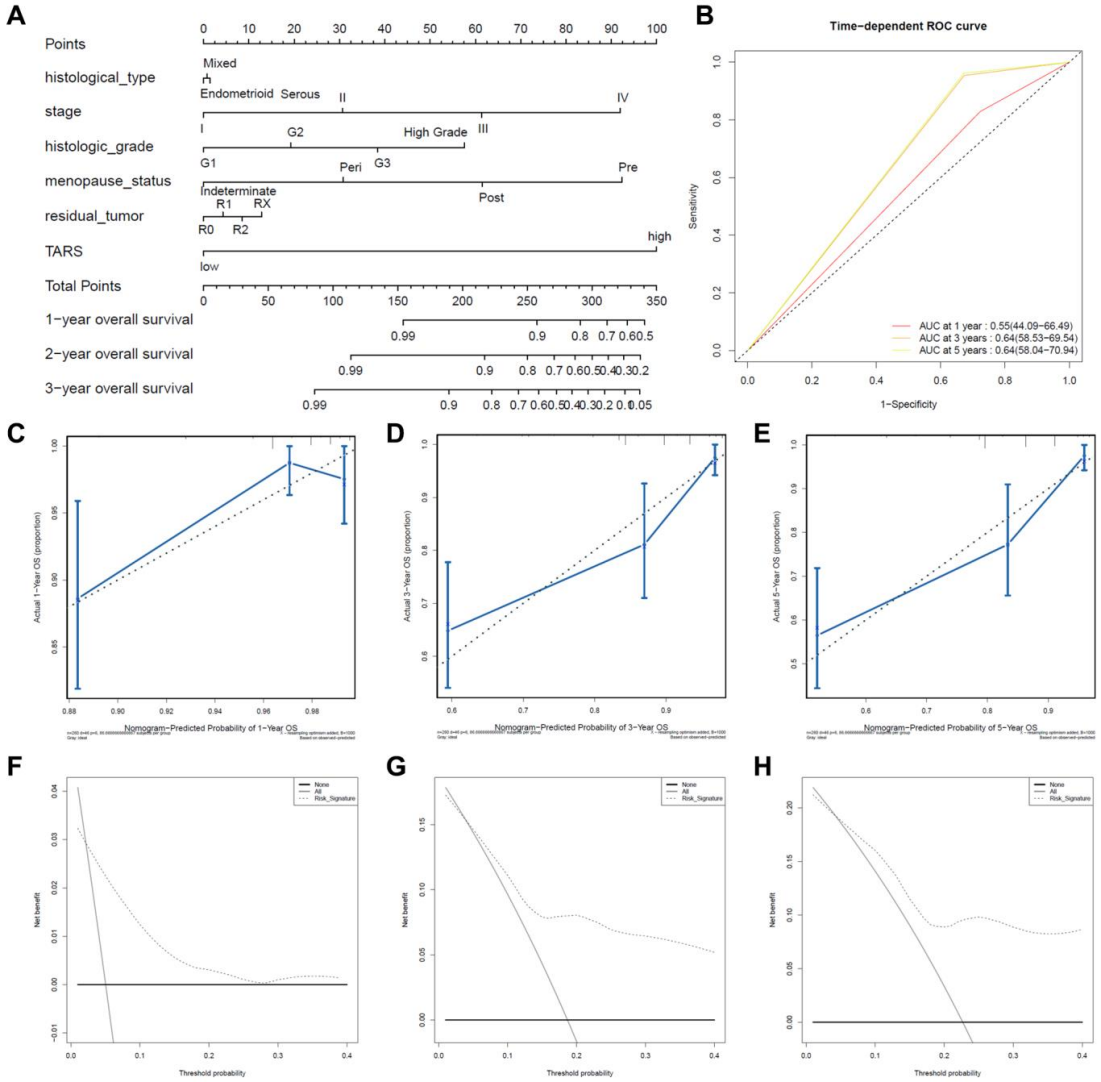
**Predictive value of TARS expression in overall survival.** (**A**, **B**) ROC curves evaluating the TARS expression for predicting overall survival. (**C**) Nomogram predicted 1-year overall survival vs. actual 1-year overall survival. (**D**) Nomogram predicted 3-year overall survival vs. actual 3-year overall survival. (**E**) Nomogram predicted 5-year overall survival vs. actual 5-year overall survival. (**F**–**H**) Decision curve analysis reflects the feasibility of TARS expression in predicting 1-year, 3-year, and 5-year overall survival.

The nomogram was used to study the predictive value of TARS expression. High TARS expression had shorter overall survival (Figure 8A). Higher stage, histologic grade, pre-menopause status, or more residual tumor exhibited shorter overall survival. The ROC curves showed moderate diagnostic capability (Figure 8B). The nomogram-predicted probability of 1-year (Figure 8C), 3-year (Figure 8D), and 5-year (Figure 8E) overall survival was close to the corresponding actual overall survival, respectively. Moreover, the decision curves reflecting the prediction model confirmed that high TARS expression could predict shorter overall survival (Figure 8F–8H).

### Predictive value of TARS expression in disease specific survival

**Figure 9:**
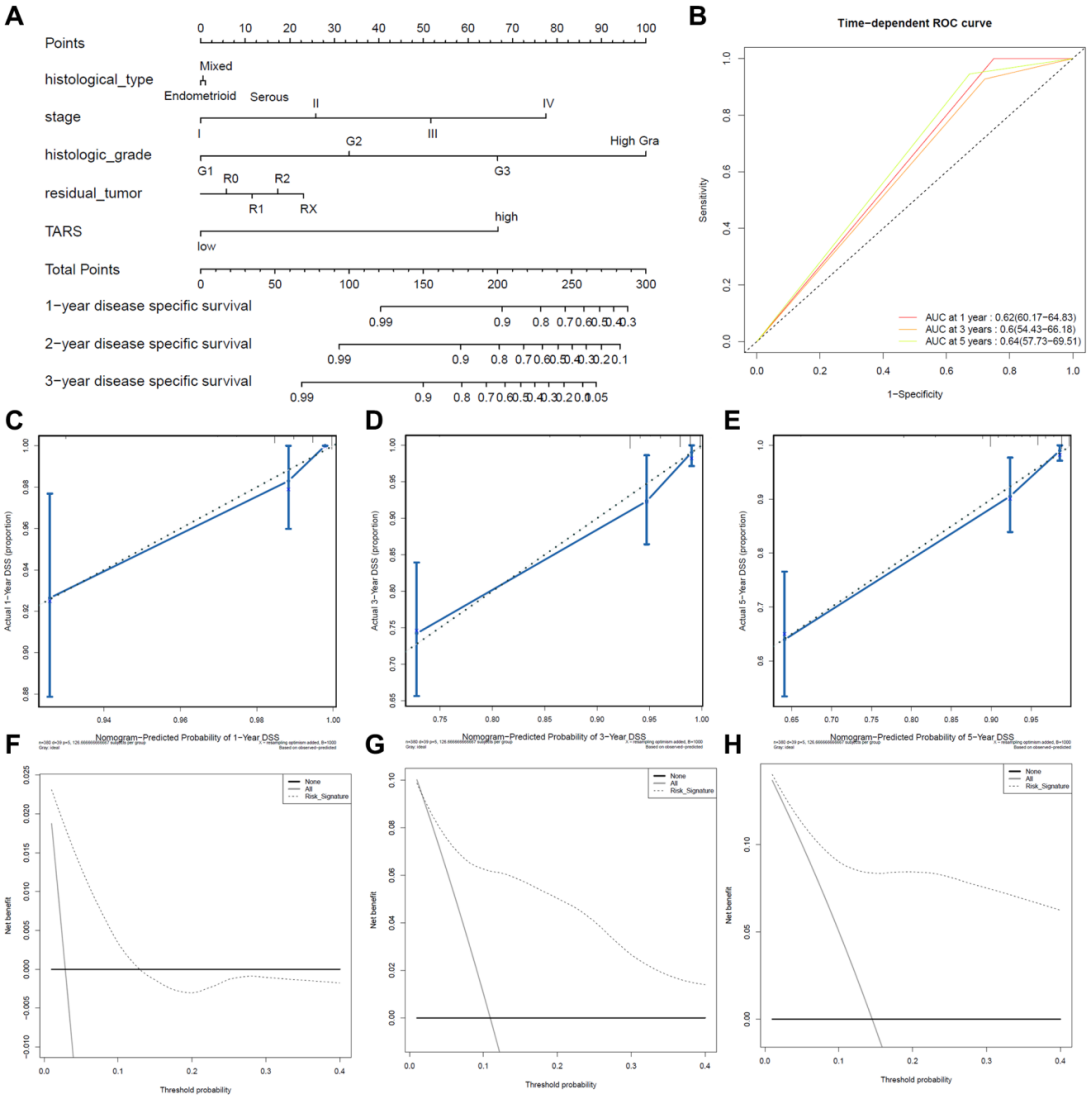
**Predictive value of TARS expression in disease specific survival.** (**A**, **B**) ROC curves evaluating the TARS expression for predicting disease specific survival. (**C**) Nomogram predicted 1-year disease specific survival vs. actual 1-year disease specific survival. (**D**) Nomogram predicted 3-year disease specific survival vs. actual 3-year disease specific survival. (**E**) Nomogram predicted 5-year disease specific survival vs. actual 5-year disease specific survival. (**F**–**H**) Decision curve analysis reflects the feasibility of TARS expression in predicting 1-year, 3-year, and 5-year disease specific survival.

High TARS expression had shorter disease specific survival (Figure 9A). Higher stage, histologic grade, or more residual tumor had shorter disease specific survival. The ROC curves showed moderate diagnostic capability (Figure 9B). The nomogram-predicted probability of 1-year (Figure 9C), 3-year (Figure 9D), and 5-year (Figure 9E) disease specific survival was close to the corresponding actual disease specific survival, respectively. Moreover, the decision curves reflecting the prediction model confirmed that high TARS expression could predict shorter disease specific survival (Figure 9F–9H).

### High TARS expression-enriched pathways

**Figure 10:**
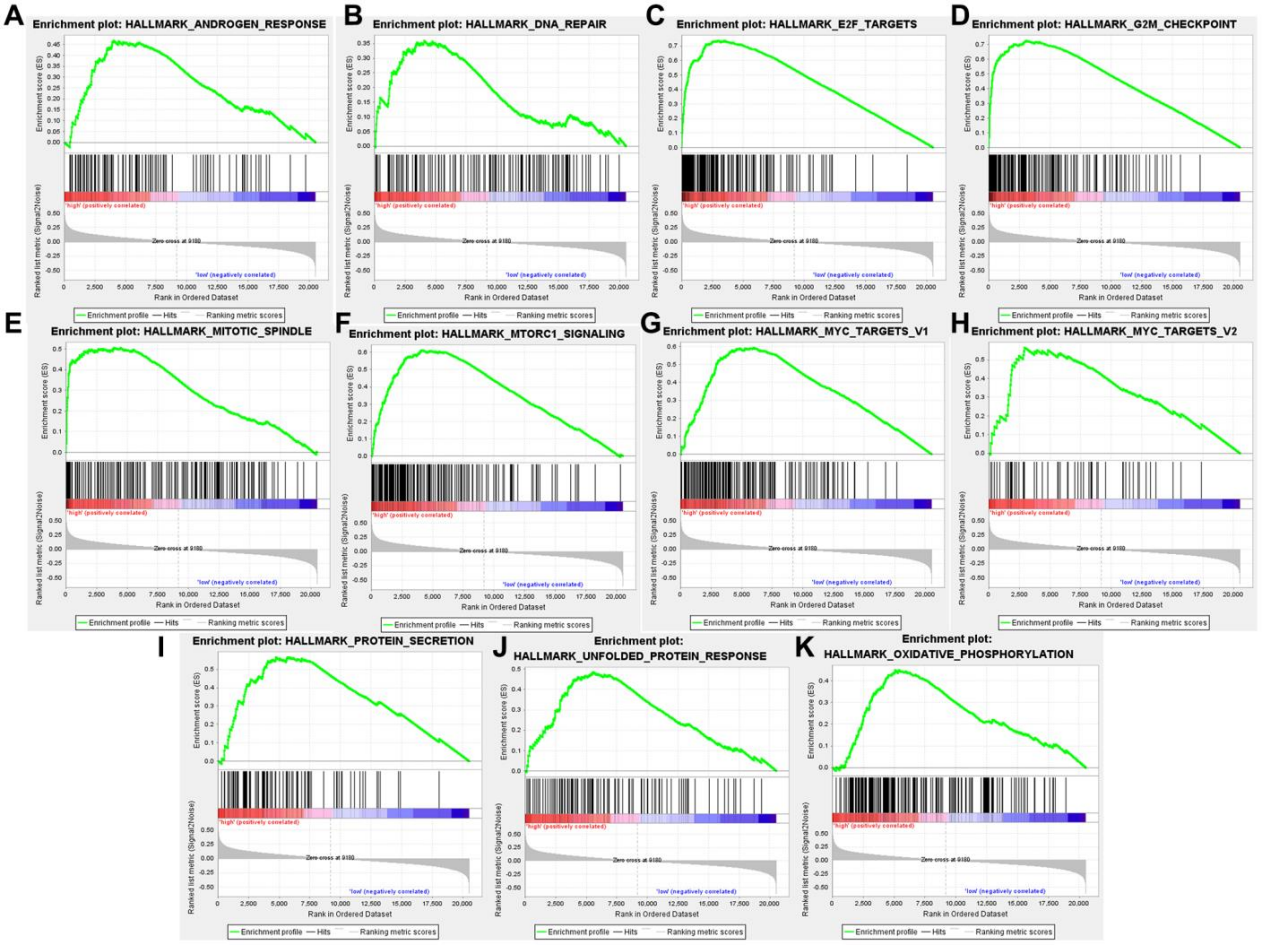
**High TARS expression-enriched pathways.** (**A**) Androgen response. (**B**) DNA repair. (**C**) E2F targets. (**D**) G2M checkpoint. (**E**) Mitotic spindle. (**F**) MTORC1 signaling. (**G**) Myc targets v1. (**H**) Myc targets v2. (**I**) Protein secretion. (**J**) Unfolded protein response. (**K**) Oxidative phosphorylation.

High TARS expression-enriched pathways were screened by GSEA analysis (Supplementary Table 2). High TARS expression was significant correlated with unfolded protein response, MTORC1 signaling, protein secretion, G2M checkpoint, mitotic spindle, Myc targets v1, DNA repair, E2F targets, oxidative phosphorylation, Myc targets v2, and androgen response (Figure 10A–10K). These high TARS expression-enriched pathways may influence the immune response and cell proliferation of endometrial cancer.

### Correlation between TARS expression and immune cells

**Figure 11:**
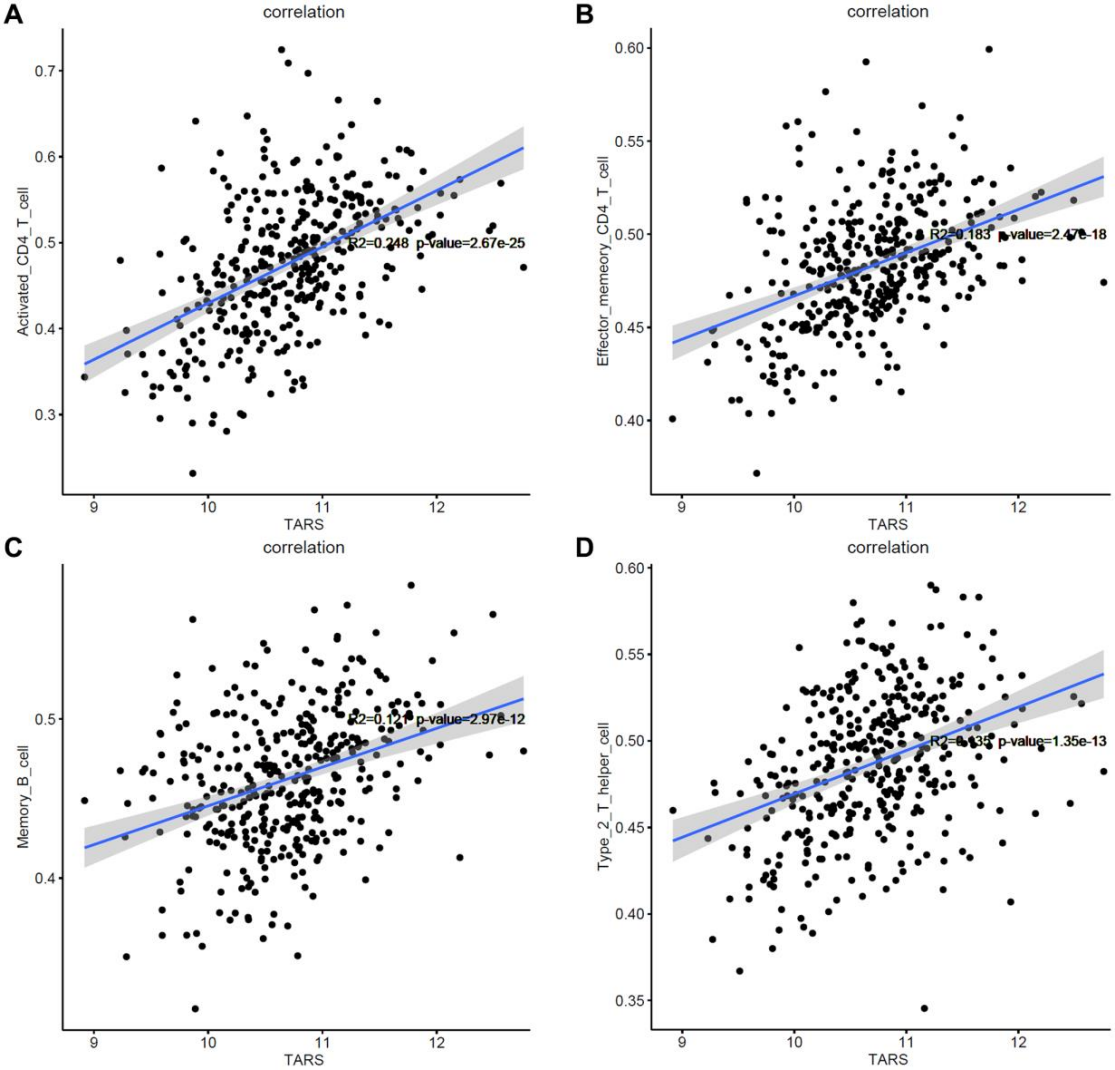
**Correlation between TARS expression and immune cells.** (**A**) Activated CD4^+^ T cell. (**B**) Effector memory CD4^+^ T cell. (**C**) Memory B cell. (**D**) Type 2 T helper cell.

Based on the results of GSEA analysis, the correlation between TARS expression and immune cells were evaluated. After screening, only 4 types of immune cells showed significant correlation with TARS expression (*P* < 0.001), including activated CD4^+^ T cell (Figure 11A), effector memory CD4^+^ T cell (Figure 11B), memory B cell (Figure 11C), and type 2 T helper cell (Figure 11D). The results suggested that these 4 types of immune cells may participate in the high TARS expression related immune response in endometrial cancer.

### High TARS expression in tissue and cell

**Figure 12:**
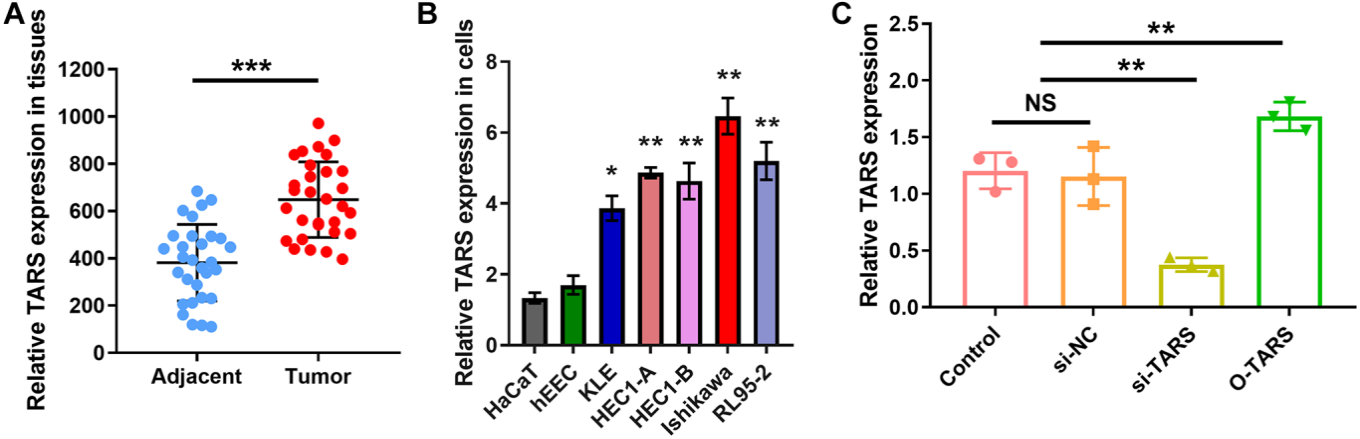
**High TARS expression in tissue and cell.** (**A**) TARS expression in 30 endometrial cancer tissues and adjacent normal tissues by qRT-PCR. (**B**) Relative TARS expression in HaCaT, hEEC, KLE, HEC1-A, HEC1-B, Ishikawa, and RL95-2 by qRT-PCR. (**C**) Relative TARS expression in Ishikawa cells transfected with control, si-NC, si-TARS, and O-TARS by qRT-PCR. Abbreviation: NS; no significance; ^**^P < 0.01; ^***^P < 0.001.

Compared with adjacent normal tissue, TRAS expression was significantly higher (*P* < 0.001) in endometrial cancer (Figure 12A). Also, significant higher TARS expression was observed in endometrial cancer cell lines (Figure 12B). Of note, Ishikawa showed the highest TARS expression, therefore used in the subsequent cell proliferation experiments. The effect of si-TRAS and O-TARS on TARS expression was verified (Figure 12C). TARS expression was significantly lower in si-TARS group (*P* < 0.01), and higher in O-TARS group (*P* < 0.01).

### TARS knockdown inhibits cell proliferation

**Figure 13:**
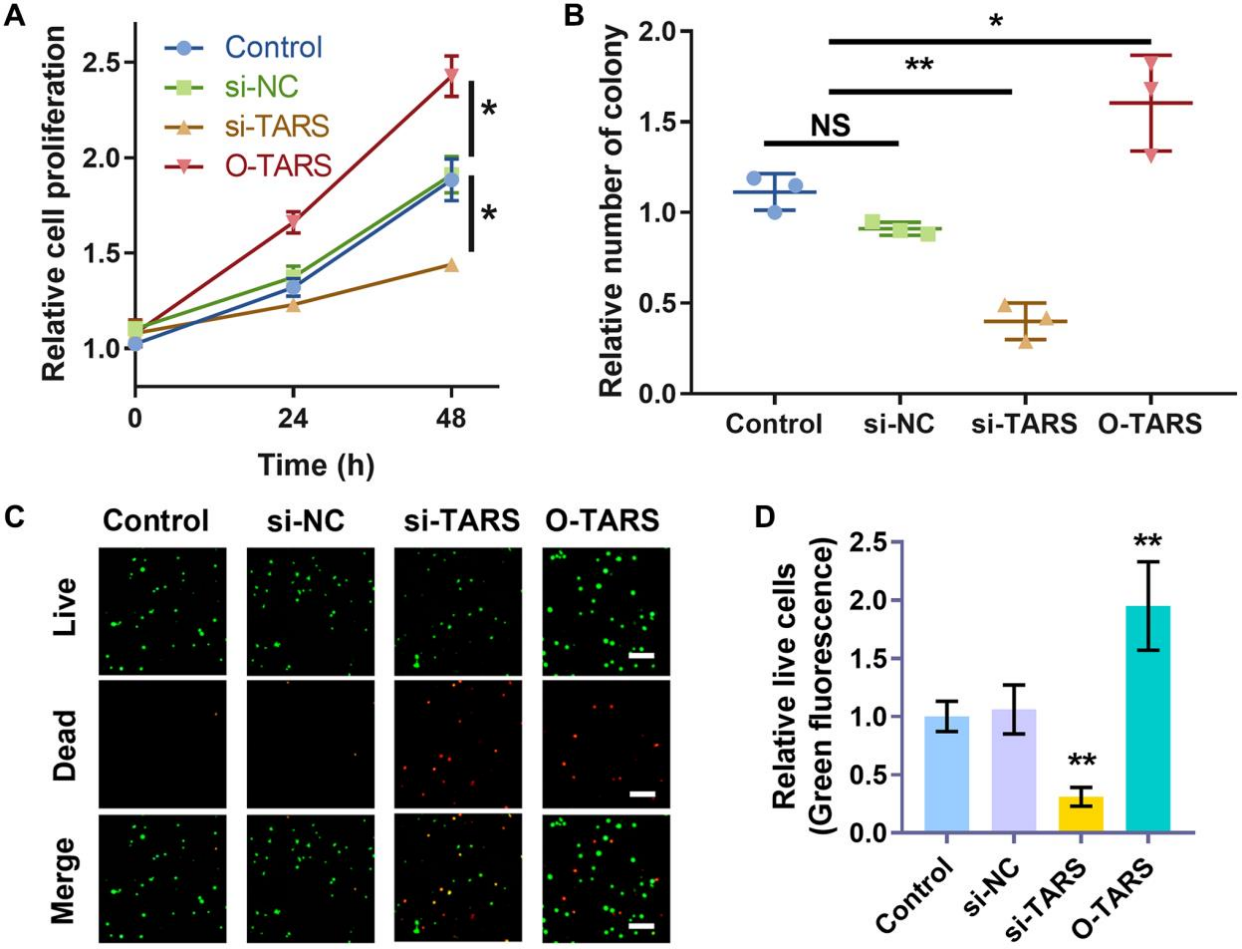
**TARS knockdown inhibits cell proliferation.** (**A**) Relative cell proliferation of Ishikawa cell by CCK-8 assay. (**B**) Relative number of colonies of Ishikawa cell. (**C**) Co-staining of calcein AM and PI of Ishikawa cell, and (**D**) relative live cells. The live cells were stained with green fluorescence, and the dead cells were stained with red fluorescence. Scale bar = 50 μm. Abbreviation: NS; no significance; ^*^P < 0.05; ^**^P < 0.01.

The function of TARS on Ishikawa cell proliferation was studied using strategies of knockdown and over expression. The CCK-8 results (Figure 13A) showed significantly inhibited cell proliferation in si-TARS (*P* < 0.05) and promoted cell proliferation in O-TARS (*P* < 0.05). Besides, the colony formation results (Figure 13B) showed decreased colonies in si-TARS (*P* < 0.01) and increased colonies in O-TARS (*P* < 0.05). Finally, the live/dead staining further confirmed the results (Figure 13C). By quantification (Figure 13D), there were fewer live cells in si-TARS (*P* < 0.01) and more live cells in O-TARS (*P* < 0.01).

## DISCUSSION

The evolution of endometrial cancer is involved in multiple genes and develops in multiple steps, which is mainly related to the activation of protooncogenes, and loss or mutation of tumor suppressor genes [22]. The cancer heterogeneity and individual differences have brought additional difficulties to the diagnosis and precise treatment. The rapid breakthrough of the whole genome sequencing technology provides new ideas for the clinical problems and the study of related pathological mechanism [23]. The bioinformatics analysis based on a few samples increases the risk of obtaining fake positive results. Therefore, this study searched and downloaded the expression data of endometrial cancer and normal endometrial tissue genes from TCGA database.

In this study, high TARS expression was found in endometrial cancer. TARS was also significantly highly expressed in serous type (*P* = 0.0011), G3 grade (*P* < 0.001), and deceased status (*P* = 0.0012). The results showed significant association between high TARS expression with poor overall survival (*P* = 0.0012) and poor disease specific survival (*P* = 0.0034). In recent years, the incidence and disease mortality of endometrial cancer have been increased around the world [24]. The pathogenesis of endometrial cancer is not clear, but it is generally believed that hypertension and diabetes are high-risk factors for endometrial cancer [25]. Here, significant differences were observed in advanced stage (*P* = 0.0053), G3 and G4 (*P* = 0.015), and old (*P* = 0.0032). Nevertheless, early stage, G1 and G2, and young subgroups showed no statistical differences. The stage (HR: 1.589, 95% CI: 1.246–2.027, *P* < 0.001), diabetes (HR: 1.556, 95% CI: 1.068–2.267, *P* = 0.021), histologic grade (HR: 2.078, 95% CI: 1.289–3.352, *P* = 0.003), and TARS expression (HR: 4.912, 95% CI: 1.765–13.674, *P* = 0.002) showed independent prognostic value for overall survival of endometrial cancer. Significant differences were observed in advanced stage (*P* = 0.0076), G3 and G4 (*P* = 0.026), and old (*P* = 0.0092). Nevertheless, early stage, G1 and G2, and young subgroups showed no statistical differences. The stage (HR: 2.109, 95% CI: 1.536–2.897, *P* < 0.001), histologic grade (HR: 2.646, 95% CI: 1.335–5.244, *P* = 0.005), and TARS expression (HR: 6.723, 95% CI: 1.606–28.152, *P* = 0.009) showed independent prognostic value for disease specific survival of endometrial cancer.

Although the diagnosis and treatment methods and prognosis of endometrial cancer have made considerable progress in recent years, the incidence and mortality of endometrial cancer have not been reduced [26]. There is an emergency need to effectively predict the prognostic indicators to improve the survival of patients with endometrial cancer [27]. However, endometrial cancer has no specific serum biomarkers, and the prognostic biomarkers are also limited [28]. Our results found high TARS expression could predict shorter overall survival and disease specific survival. High TARS expression was confirmed in tissue and cell.

Biomarkers are valuable for screening women with high risk of endometrial cancer, dividing patients into different prognosis risks, and evaluating the prognosis differences to achieve personalized treatment [29, 30]. The tumor biomarker CA125 (Carbohydrate Antigen 125) contributes to the diagnosis of endometrial cancer [31, 32]. Compared with early endometrial cancer, the concentration of CA125 usually increases in type II or advanced endometrial cancer [33]. The 5-year survival rate of endometrial cancer metastasis dropped to 17%. So far, there are no biomarkers with high specialty and strong sensitivity as an early diagnosis or prognostic evaluation indicator [34]. In our study of TARS, the AUC of ROC curve between normal and tumor was 0.901. Besides, the AUC was 0.890 for stage I, 0.864 for stage II, 0.936 for stage III, and 0.957 for stage IV. The results indicated promising diagnostic value of TARS expression.

High TARS expression was significant correlated with unfolded protein response, MTORC1 signaling, protein secretion, G2M checkpoint, mitotic spindle, Myc targets v1, DNA repair, E2F targets, oxidative phosphorylation, Myc targets v2, and androgen response. The results suggested that activated CD4^+^ T cell, effector memory CD4^+^ T cell, memory B cell and type 2 T helper cell may participate in the high TARS expression related immune response in endometrial cancer. Autoantibodies directed against one or more ARSs are present in anti-synthetase syndrome (ASSD), an autoimmune illness that is also defined by clinical symptoms [35]. Zhou et al. reported the tumor mutation burden and immune infiltrates in endometrial cancer [36].

The function of TARS on Ishikawa cell proliferation was studied using strategies of knockdown and over expression. The CCK-8 results showed significantly inhibited cell proliferation in si-TARS (*P* < 0.05) and promoted cell proliferation in O-TARS (*P* < 0.05). Besides, the colony formation results showed decreased colonies in si-TARS (*P* < 0.01) and increased colonies in O-TARS (*P* < 0.05). Finally, the live/dead staining further confirmed the results. By quantification, there were fewer live cells in si-TARS (*P* < 0.01) and more live cells in O-TARS (*P* < 0.01). Studies of Vivacqua et al. have found that active metabolites of selective estrogen receptor modulator TAM, 4-hydroxyl Moqifen, promotes the cell proliferation of Ishikawa and HEC1-A through the GPR30 pathway rather than rely on ERα's rapid response pathway [37]. The molecular mechanisms of TARS in immune response and cell proliferation need to be further explored. The study is in lack of prospective follow-up data for further verification.

## CONCLUSION

High TARS expression was found in endometrial cancer with prognostic and predictive value. High TARS expression is significantly associated with poor overall survival and poor disease specific survival. Activated CD4^+^ T cell, effector memory CD4^+^ T cell, memory B cell, and type 2 T helper cell may participate in the high TARS expression related immune response in endometrial cancer. TARS knockdown inhibits cell proliferation. This study will provide new biomarker TARS for diagnosis and prognosis of endometrial cancer.

## Supplementary Materials

Supplementary Figure 1

Supplementary Tables
