# Supplementary material for: High expression of TARS is associated with poor prognosis of endometrial cancer
Source: Aging (Albany NY). 2023 Mar 6;15(5):1524–42. doi: 10.18632/aging.204558 (PMC10042687; doi:10.18632/aging.204558)
Supplement: Supplementary Figure 1 [file aging-15-204558-s001.pdf]

SUPPLEMENTARY FIGURE

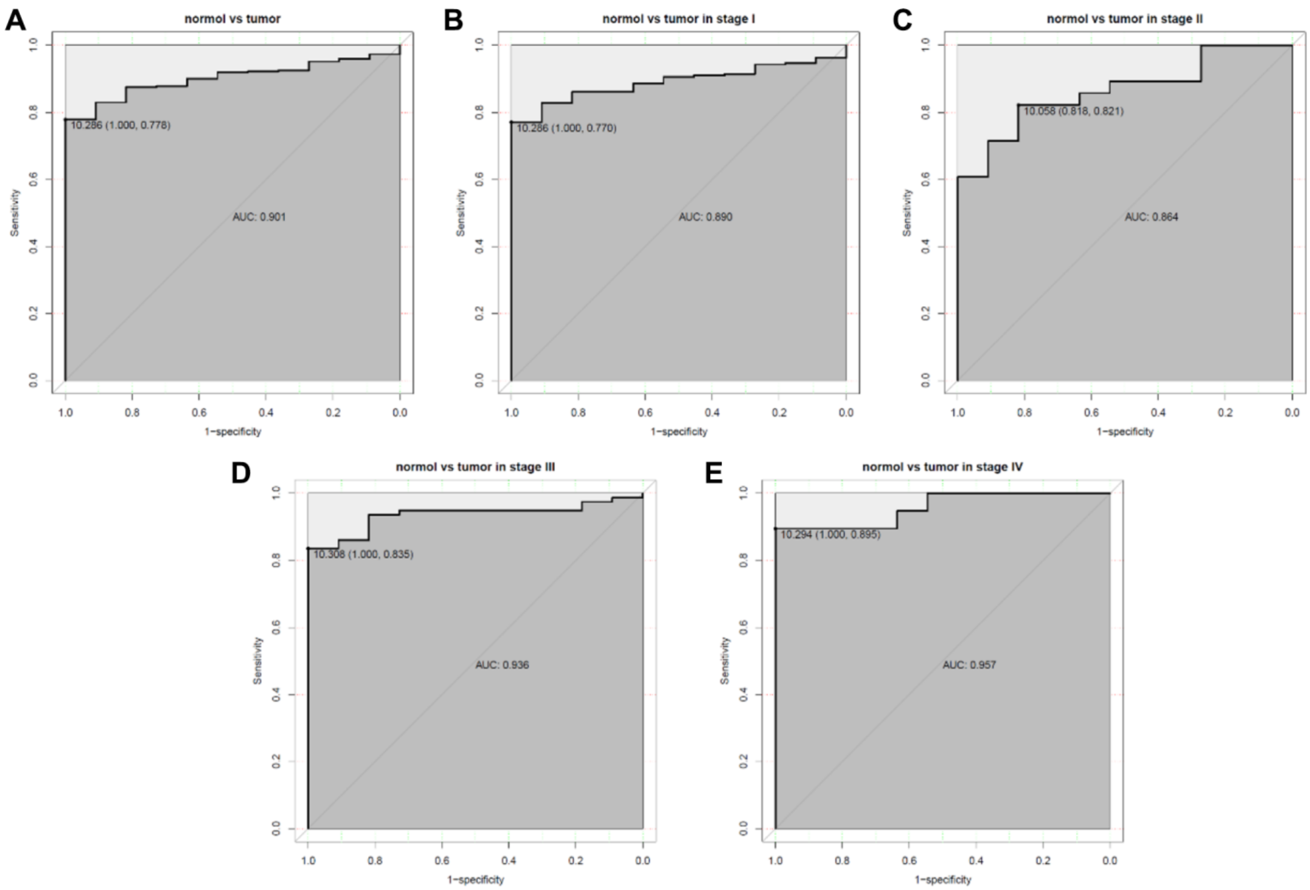

Supplementary Figure 1. Diagnostic value of TARS expression. ROC curve in (**A**) normal vs. tumor, (**B**) normal vs. tumor in stage I, (**C**) normal vs. tumor in stage II, (**D**) normal vs. tumor in stage III, and (**E**) normal vs. tumor in stage IV.
