# Supplementary material for: High expression of TARS is associated with poor prognosis of endometrial cancer
Source: Aging (Albany NY). 2023 Mar 6;15(5):1524–42. doi: 10.18632/aging.204558 (PMC10042687; doi:10.18632/aging.204558)
Supplement: Supplementary Tables [file aging-15-204558-s002.pdf]

SUPPLEMENTARY TABLES

Supplementary Table 1. Characteristics of patients with endometrial cancer.

**Parameter**		***N***	**High**	**(%)**	**Low**	**(%)**	**χ^2**	***P***
Age	<55	72	57	(19.66)	15	(18.75)	0.0005	0.9828
	≥55	298	233	(80.34)	65	(81.25)		
Histological type	Endometrioid	303	235	(81.03)	68	(85.00)	1.0990	0.5773
	Mixed	10	9	(3.10)	1	(1.25)		
	Serous	57	46	(15.86)	11	(13.75)		
Stage	I	244	190	(65.52)	54	(67.50)	7.8170	0.0662
	II	28	17	(5.86)	11	(13.75)		
	III	79	66	(22.76)	13	(16.25)		
	IV	19	17	(5.86)	2	(2.50)		
Diabetes	No	209	153	(68.61)	56	(80.00)	2.8459	0.0916
	Yes	84	70	(31.39)	14	(20.00)		
Hypertension	No	127	100	(41.15)	27	(37.50)	0.1748	0.6759
	Yes	188	143	(58.85)	45	(62.50)		
Histologic grade	G1	85	60	(20.69)	25	(31.25)	14.2642	**0.0015**
	G2	100	72	(24.83)	28	(35.00)		
	G3	178	154	(53.10)	24	(30.00)		
	High Grade	7	4	(1.38)	3	(3.75)		
Menopause status	Indeterminate	14	10	(3.60)	4	(5.26)	1.9898	0.5907
	Peri	14	11	(3.96)	3	(3.95)		
	Post	300	234	(84.17)	66	(86.84)		
	Pre	26	23	(8.27)	3	(3.95)		
Residual tumor	R0	265	200	(81.97)	65	(89.04)	2.8421	0.5075
	R1	18	14	(5.74)	4	(5.48)		
	R2	11	10	(4.10)	1	(1.37)		
	Rx	23	20	(8.20)	3	(4.11)		
Vital status	Deceased	58	55	(18.97)	3	(3.75)	9.8611	**0.0017**
	Living	312	235	(81.03)	77	(96.25)		
TARS	High	290	290	(100.00)	0	(0)	364.1227	**<0.001**
	Low	80	0	(0)	80	(100.00)		

X, unknown.

Supplementary Table 2. High TARS expression-enriched pathways.

**Name**	**Size**	**ES score**	**NES score**	***P* value**	***Q* value**
Unfolded protein response	113	0.4861	2.4835	<0.001	<0.001
MTORC1 signaling	198	0.6119	2.4538	<0.001	<0.001
Protein secretion	96	0.5664	2.4074	<0.001	<0.001
G2M checkpoint	194	0.7253	2.0167	0.0019	0.0200
Mitotic spindle	199	0.5071	1.9840	0.0060	0.0202
Myc targets v1	195	0.5939	1.9714	0.0124	0.0189
DNA repair	149	0.3603	1.9352	0.0242	0.0207
E2F targets	193	0.7381	1.8950	0.0020	0.0252

Oxidative phosphorylation	199	0.4494	1.8859	0.0296	0.0230
Myc targets v2	58	0.5656	1.8186	0.0390	0.0319
Androgen response	101	0.4677	1.7529	0.0020	0.0456
